# *SOD1* Gene Silencing Promotes Apoptosis and Suppresses Proliferation of Heat-Stressed Bovine Granulosa Cells via Induction of Oxidative Stress

**DOI:** 10.3390/vetsci8120326

**Published:** 2021-12-13

**Authors:** Adnan Khan, Muhammad Zahoor Khan, Jinhuan Dou, Huitao Xu, Lei Liu, Huabin Zhu, Yachun Wang

**Affiliations:** 1 Key Laboratory of Animal Genetics, Breeding, and Reproduction, MARA, National Engineering Laboratory for Animal Breeding, College of Animal Science and Technology, China Agricultural University, Beijing 100193, China; dr.adnan93@cau.edu.cn (A.K.); zahoorcau@cau.edu.cn (M.Z.K.); doujinhuan@cau.edu.cn (J.D.); 2 Shenzhen Branch, Guangdong Laboratory of Lingnan Modern Agriculture, Genome Analysis Laboratory of the Ministry of Agriculture and Rural Affairs, Agricultural Genomics Institute at Shenzhen, Chinese Academy of Agricultural Sciences, Shenzhen 518124, China; liulei03@caas.cn; 3 State Key Laboratory of Animal Nutrition, Beijing Engineering Technology Research Center of Raw Milk Quality and Safety Control, College of Animal Science and Technology, China Agricultural University, Beijing 100193, China; 4 Embryo Biotechnology and Reproduction Laboratory, Institute of Animal Sciences, Chinese Academy of Agricultural Sciences, Beijing 100193, China; xuhuitao104@163.com (H.X.); zhuhuabin@caas.cn (H.Z.)

**Keywords:** bovine granulosa cells, heat stress, *SOD1* silencing, oxidative stress, apoptosis, cell proliferation

## Abstract

Heat stress (HS) compromises dairy cattle reproduction by altering the follicular dynamics, oocyte maturation, and normal physiological function of ovarian granulosa cells (GCs), eventually resulting in oxidative damage and cell apoptosis. To protect the cells from oxidative damage, the Superoxide dismutase-1 (*SOD1*) degraded the hydrogen peroxide (H_2_O_2_) to oxygen (O_2_) and water. The objective of the current study was to investigate the impact of *SOD1* silencing on intracellular ROS accumulation, cell viability, MMP, hormone synthesis (P4, E2), cell proliferation, and apoptosis in GCs under HS. The mechanistic role of *SOD1* regulation in the heat-stressed GCs was explored. *SOD1* gene was successfully silenced in GCs and confirmed at both transcriptional and translational levels. We found that silencing of *SOD1* using *si*RNA under HS aggravated intracellular accumulation of reactive oxygen species, apoptosis, disrupted the mitochondrial membrane potential (MMP), altered transition of the cell cycle, and impaired synthesis of progesterone (P4) and estrogen (E2) in GCs. The associative apoptotic, steroidogenic, and cell cycle genes (*BAX*, *Caspase-3*, *STAR*, *Cyp11A1*, *HSP70*, *PCNA*, and *CyclinB1*) were used to confirm the results. These results identify a novel role of *SOD1* in the modulation of bovine ovarian GC apoptosis, which provides a target for improving the fertility of heat-stressed dairy cows in summer.

## 1. Introduction

Environmental temperature significantly affects animal breeding and reproduction [1]. Heat stress causes infertility in dairy cows, a significant source of economic loss in the cattle industry [2,3,4,5]. Loses related to HS due to decreased milk production and a decline in pregnancy rates cost U.S. producers about one billion dollars annually [6]. The ovarian follicle is composed of oocytes surrounded by several layers of GCs. GCs play an important role in supporting and nurturing the developing oocyte. Subsequently, the matured oocyte further participates in the fertilization and formation of an embryo [7]. Heat stress has long been considered a challenging issue that negatively affects the reproductive functions of dairy cattle, altering the developmental competence of oocytes during fetal growth [8]. Moreover, the conception rate in dairy cows is lower if there are five weeks of HS before breeding [9,10]. Likewise, heat-tolerant Gir (*Bos indicus*) cows have less follicular growth, oocyte developmental competence, and follicular steroidogenic ability, with these effects continuing to exist for more than 100 days even after the end of the HS period [3].

The ovarian pool of follicles and their enclosed GCs and oocytes are highly susceptible to HS among the female reproductive system contents [8]. Follicular development is highly dependent on the function of GCs. The follicular GCs have an important role in oocyte nurturing and hormonal synthesis, facilitating functional crosstalk with the developing oocyte [10,11]. The synthesis of two main reproductive hormones (E2 and P4) is one of the major roles of GCs [12,13]. Ovarian follicular development, oocyte maturation, and proliferation of endometrium are highly dependent on the production of E2 by GCs [14]. However, HS compromises the normal physiology and functions of GCs. Heat stress is well known to promote the intracellular accumulation of ROS such as H_2_O_2_, superoxide radical (O_2_), and hydroxyl radicals (OH) abundance in GCs, thereby causing oxidative stress [15,16] and apoptosis [17]. Cellular redox status plays a critical role in cell survival, growth, and death signaling. At the same time, an overwhelming increase in intracellular ROS due to HS could cause a series of damage to GCs, including apoptosis, altered cell proliferation, disruptive MMP, and impaired synthesis of E2 and P4 [18,19,20,21].

To combat oxidative stress, cells express various antioxidant enzymes such as superoxide dismutases (*SOD*s), glutathione peroxidases, and catalases (*CAT*). *SOD*s are well known for mediating the dismutation of the superoxide radicals into O_2_ and H_2_O_2_; hence play an important role against oxidative stress [22,23]. Therefore, *SOD*s are considered to be the very first line of defense against ROS [22]. *SOD1* and *SOD2* are two distinct intracellular *SOD* isoenzymes [24]. *SOD1* is a Cu/Zn enzyme mostly expressed in the cytosol, with a little amount in mitochondrial intermembrane space. [25,26,27].

Moreover, many studies have explored the regulation of *SOD1* in GCs under HS, but no attempt has been taken so far to functionally validate the impact of *SOD1* gene silencing on GCs functions under HS. Our previous study on transcriptomic analysis of GCs under HS showed a significant upregulation of the *SOD1* gene [16]. For instance, the current study was aimed to explore the role of *SOD1* on intracellular ROS accumulation, cell viability, MMP, hormone synthesis (P4, E2), cell proliferation, and apoptosis in GCs under HS. For instance, we silenced the *SOD1* gene in GCs under HS. Recently, RNA interference (RNAi) has been proven to be able to modulate and modify a particular gene knockdown model in many species [28]. After successful transfection, GCs were examined for ROS abundance, cell viability, MMP, hormone synthesis, cell proliferation, and apoptosis. We found that *SOD1* has an essential role in the regulation of these parameters under HS.2.

## 2. Materials and Methods

### 2.1. Ethical Approval

This study protocols for collecting bovine ovaries from experimental animals were reviewed and approved by the institutional animal care and use committee of China Agricultural University Beijing, China (permit number: DK996).

### 2.2. The Isolation, Culture, Identification, and Treatment of Granulosa Cells

All techniques involved in ovaries collection from Holstein cattle, isolation of GCs, identification of GCs by immunofluorescence staining, and cell culture were performed using a previously described procedure [29]. After culturing primary GCs for 48 h, different treatment conditions were applied to the GCs. To construct the HS model, three parallel groups were set up: 38 °C + negative control (NC), 40 °C + NC, and 40 °C + small interfering *SOD*1 (*siSOD1*; that silences the *SOD1* gene). The cells were then grown for 24 h at 38 °C. After the cultivation, the cells and culture media were collected for further analysis.

### 2.3. Production of siRNA and GCs Transfection

RNA interference was carried out for *SOD1* silencing using small interfering RNAs directed against cow *SOD1* (*siSOD1*) (the sense and antisense sequence of siRNA are 5′-CCAUCAGUUUGGAGACAAUTT-3′, 5′-AUUGUCUCCAAACUGAUGGTT-3′, respectively) and negative control (NC) (Gene Pharma, Shanghai, China). Bovine GCs were cultured in six-well plates for 48 h until 60% confluence and then transfected with LipofectamineTM 3000 (Invitrogen, Carlsbad, ON, Canada) following the manufacturer’s instructions. Subsequently, cells were immediately suspended in DMEM/F-12 (Gibco, Life Technologies Inc., Grand Island, NY, USA) medium and incubated at 38 °C under 5% CO_2_ in humidified air. After 24–48 h of transfection, GCs were collected for protein and RNA extraction to validate the effective reduction of *SOD1* expression through western blotting and RT-qPCR. In addition, culture media was taken to estimate hormone levels.

### 2.4. Quantitative Reverse Transcription PCR (RT-qPCR)

Total RNA was extracted from GCs using an RNA kit (Tiangen, Beijing, China). RNA concentration determined using NanoDrop 2000 spectrophotometer (Thermo Scientific, Waltham, MA, USA). The expression of the selected genes was quantified through the real-time PCR analysis using iTaq™ Universal SYBR^®^ Green Supermix (Bio-Rad Laboratories GmbH, Munich, Germany) in applied Biosystem^®^ StepOnePlus™ (Applied biosystems, CA, USA). The *GAPDH* was kept as a housekeeping gene to normalize the relative expression of target genes, i.e., *SOD1*, Bcl-2 associated x-protein (*BAX*), *Caspase-3*, proliferating cell nuclear antigen (*PCNA*), *CyclinB1*, heat shock protein 70 (*HSP70*), Steroidogenic acute regulatory protein (*STAR*), and Cytochrome, P450, family 11, Subfamily A, polypeptide 1 (*Cyp11A1*). To design gene-specific primers, the Primer3, web version 4.0.0 (http://bioinfo.ut.ee/primer3/ accessed on 12 November 2021) and primer-Blast (http://www.ncbi.nlm.nih.gov/tools/primer-blast/ accessed on 12 November 2021) were used (Table 1). Data was collected using the second derivative maximum method and then analyzed further. The gene expression levels were calculated using the 2-CT technique [30].

### 2.5. Protein Extraction and Western Blotting

RIPA lysis buffer (Beyotime, Shanghai, China) containing proteinase inhibitors was used to lyse bovine GCs from each group. The extracted protein content was determined using a BCA Protein Assay Kit (Beyotime). Samples containing 50 μg were separated on sodium dodecyl sulfate-polyacrylamide gels (SDS-PAGE, 12% acrylamide gel containing 0.1% SDS), and transferred onto a polyvinylidene difluoride (PVDF) membrane (BioTraceNT, Pall Corp., Port Washington, NY, USA). Membranes were then blocked for 1 h at 37 °C with 5% (*w*/*v*) skim milk in Tris-buffered saline (TBS) with 0.1% Tween 20 (TBST). Primary antibodies against *SOD1* (CST 2770S), *BAX* (CST2772S), *Caspase-3* (CST 9662S), *PCNA* (CST 81628S), *CyclinB1* (CST 60691S), *STAR* (ab237908), *Cyp11A1* (ab175408), *HSP70* (CST 4872S), and β-actin (CST 4967S) were incubated overnight at 4 °C on the membranes (Cell Signaling Technology, Beverly, MA, Abcam, USA). The membranes were then washed three times with TBST and incubated at room temperature for one hr with HRP-conjugated secondary antibody (Zhongshan Biotechnology, Beijing, China). Finally, the protein bands were visualized using enhanced chemiluminescence (ECL) detection kit (Tanon, Shanghai, China). Proteins were quantified by densitometry using Image J 1.44p software, and β-actin was used as loading controls for normalization.

### 2.6. Estimation ROS

ROS was estimated in heat stress GCs following the protocol, previously carried out by [28]. GCs from the treated and control groups ((NC, 40 °C + NC, and *siSOD1* + 40 °C) were trypsinized and collected 48 h after culturing to study the net intracellular ROS production using a fluorescence microscope (Olympus, Tokyo, Japan). The GCs were rinsed in PBS after treatment, and 10 mol/L H2DCFDA was added to each well. GCs were washed once in 0.1% PVA/DPBS and inspected under a fluorescence microscope after incubation for 30 min at 38 °C in the dark (Olympus, Tokyo, Japan).

### 2.7. Estimation of Apoptotic and Dead GCs

Bovine GCs were detected for apoptosis using the Annexin V-FITC kit (Beyotime Biotechnology, China). GCs were extracted and washed three times with pre-heated PBS after receiving the relevant treatments. Following collection, GCs were incubated with Annexin VFITC for 20 min in the dark and propidium iodide (PI) for 2 min, followed by flow cytometry (BD Biosciences, CA, USA). The apoptotic rate is expressed as the sum of the percentage of early (Annexin V+/PI-) and late (Annexin V+/PI-) apoptosis cells. Furthermore, the number of dead cells was determined under a laser-scanning confocal microscope (TCS SP8, Leica, Germany). FlowJo software (version Win 64-10.4.0) was used to analyze the flow cytometry data (version Win 64-10.4.0).

### 2.8. Analysis of Cell Cycle

The GCs cell cycle was detected using a cell cycle and apoptosis analysis kit (Beyotime, China). From each treated group, the GCs were harvested in a 15 mL Falcon tube (Thermo Fisher Scientific, Germany), followed by centrifugation at 750× *g* for 7 min and washing twice with 1x PBS. A minimum of 1 × 10^6^ cells was fixed in pre-chilled 70% ethanol at 4 °C overnight. Ethanol was then removed using centrifugation, and the GCs pellets were washed twice with 500 µL of 1× PBS. Cellular DNA was stained with 50 µg/mL of PI and 50 µg/mL of RNase, incubated for 30 min at 37 °C in the dark, and instantly processed in FACS Calibur (BD Biosciences, CA, USA). The percentage of cells in each cell division phase (G0–G1, S, and G2–M) was estimated using data received from the FL2-A channel using ModFit LT Version 4.1 software (http://www.vsh.com/products/mflt/index.asp accessed on 12 November 2021).

### 2.9. Assessment of Mitochondrial Membrane Potential

Briefly, to examine the MMP of GCs in all treated groups, the mitochondrial membrane potential assay kit with JC-1 (Beyotime, China) was used according to the manufacturer’s instructions. Enzymatic digestion using trypsin was used to harvest the GCs and washed with warm PBS. After collection, the MMP assay kit with JC-1 (Beyotime, China) was used to stain GCs. Flow cytometry was used to count labeled cells using a fluorescence activating cell sorter (FACS) and a Calibur flow cytometer (BD Biosciences, CA, USA). The data was analyzed using FlowJo software (version Win 64-10.4.0).

### 2.10. Cell Viability Assay

The viability of bovine GCs from all treatment groups was determined using the MTT cell proliferation and cytotoxicity test kit (njjcbio, Nanjing, China). In brief, the detached cells were inoculated into 96-well plates, and after the indicated treatments, a 50 µL 1 × MTT solution was added to each well and incubated for 4 h. After 4 h, the solution was replaced with 150 µL DMSO, and the absorbance was measured at 570 nm using ELX microplate reader (BioTek, Winooski, VT, USA).

### 2.11. E2 and P4 Levels Determination

To estimate E2 and P4 levels, the cell culture media from all groups (NC, 40 °C + NC, and *siSOD1*) were used. In addition, enzyme-linked immunosorbent (ELISA) kits for P4 and E2 (ENZO life sciences, Germany) were used to estimate their concentrations, according to the manufacturer’s instructions.

### 2.12. Statistical Analysis

All data were expressed as mean ± SEM. In each group, three biological replicates (*n* = 3) of GCs were used. SPSS 16.0 and GraphPad Prism5 software were used for statistical analysis (GraphPad Software Inc, SanDiego, CA). A one-way ANOVA was used to compare the differences between the control and treatment groups, followed by a multiple comparisons post hoc test. At *p* < 0.05, differences were judged statistically significant.

## 3. Results

### 3.1. Identification of GCs

**Figure 1:**
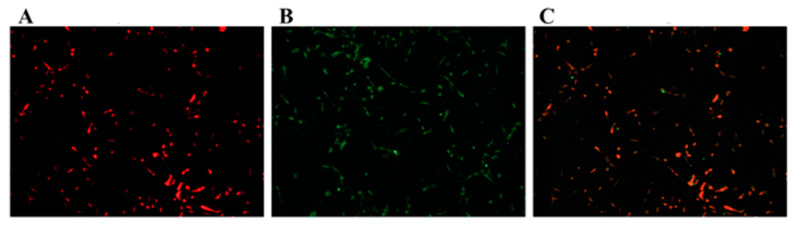
Identification of ovarian GCs by immunofluorescence. (**A**) Propidium iodide (PI) positively stained nuclei. (**B**) FSHR positive cells. (**C**) the merge of (**A**,**B**). More than 90% of the cells in the isolated, cultured cells were granulosa cells depicted that the purity of GCs was above 90%. For magnification, a 100 µm scale bar was used.

A multitude of cells makes up the ovarian follicle (GCs, theca cells etc.). Since our study focuses on bovine GCs, immunofluorescence microscopy was used to separate GCs from the rest of the follicular cells. Propidium iodide (PI) was used to stain the GCs, or an anti-FSHR antibody was used to incubate them. According to our findings, the GCs tested positive for FSHR (Figure 1).

### 3.2. Efficacy of SOD1 Transfection

**Figure 2:**
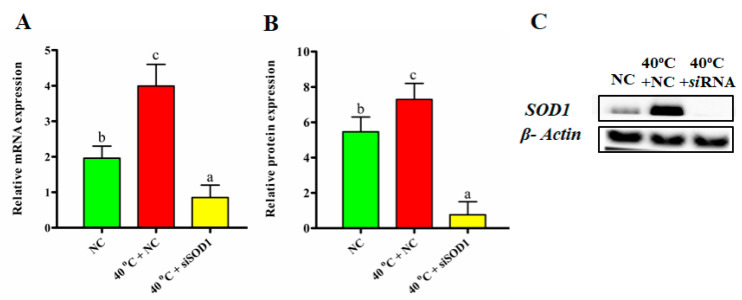
Silencing of *SOD1* gene in GCs. Transcription levels of the *SOD1* gene in all treatment groups after transfection (**A**). Western blotting was used to detect *SOD1* protein expression in GCs (**B**,**C**). The data are presented as the mean ± SEM of *n* = 3. The bars with totally distinct lettering show a statistically significant difference (*p* < 0.05).

*SOD1* was silenced with the help of *siSOD1*. After successful transfection, the GCs were separated into three groups (NC, 40 °C + NC, and 40 °C + *siSOD1*). *SOD1* expression was measured at both the translational and transcriptional levels in all groups. For the GCs transfected with *siSOD1*, RTqPCR revealed that *SOD1* mRNA expression was significantly (*p* < 0.05) lower (Figure 2A). Likewise, western blot results showed a similar protein expression depicting that *SOD1* was successfully silenced with *siSOD1* (Figure 2B,C).

### 3.3. Silencing of SOD1-Induced Intracellular ROS Accumulation under Heat Stress

**Figure 3:**
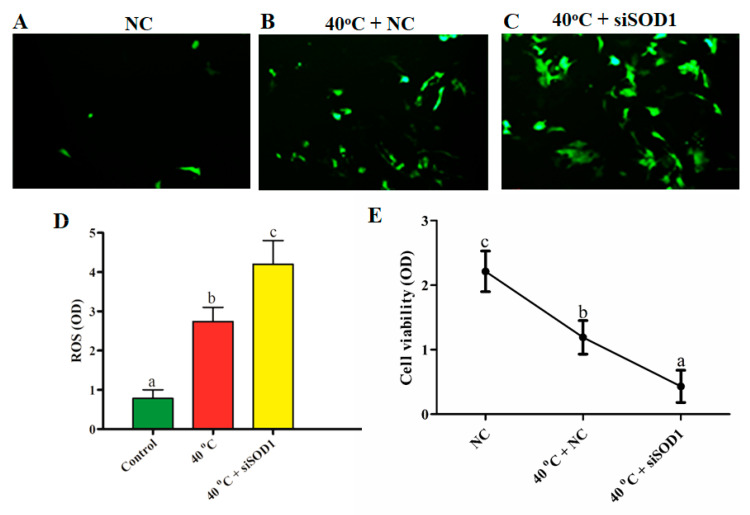
Under heat stress, *SOD1* regulates the intracellular buildup of ROS and the viability of GCs. NC (**A**) and HS (40 °C + NC, 40 °C + *siSOD1*) intracellular ROS production assessed by DCF fluorescence (**B**,**C**). Analysis of relative fluorescence emission in a quantitative manner (**D**). For magnification, a 100 µm scale bar was used. The cells transfected with siRNA under HS were used for the MMT cell viability assay (**E**). The bars with totally distinct lettering show a statistically significant difference (*p* < 0.05). Optical density (OD), Negative control (NC).

The oxidation sensitive probe 6-carboxy-2′,7′-H2DCF-DA was utilized to examine the intracellular accumulation of ROS in all groups (NC, 40 °C + NC, and 40 °C +*siSOD1*). The results showed that the intracellular abundance of ROS in the 40 °C + NC group was considerably (*p* < 0.05) higher than in the NC group. Likewise, compared with 40 °C + NC group, the fluorescence of intracellular ROS production significantly (*p* < 0.05) increased in 40 °C + *siSOD1* group. Furthermore, our results demonstrated that *SOD1* silencing induced intracellular ROS production (Figure 3A–D).

### 3.4. Silencing of SOD1-Altered Viability of GCs under Heat Stress

The viability of GCs was measured through MTT assay. It was documented that the viability of GCs in the 40 °C + NC group was significantly (*p* < 0.05) lower than that of the NC group. Similarly, a significant decline in cell viability was noted in 40 °C + *siSOD1* group than in NC and 40 °C + NC groups (Figure 3E). As a result of the oxidative stress generated by ROS production under HS, the viability of GCs was affected, and *SOD1* silencing worsened the viability of GCs, demonstrating its importance in the regulation of cell viability.

### 3.5. Silencing of SOD1 Promoted Apoptosis and Cell Death in GCs under Heat Stress

**Figure 4:**
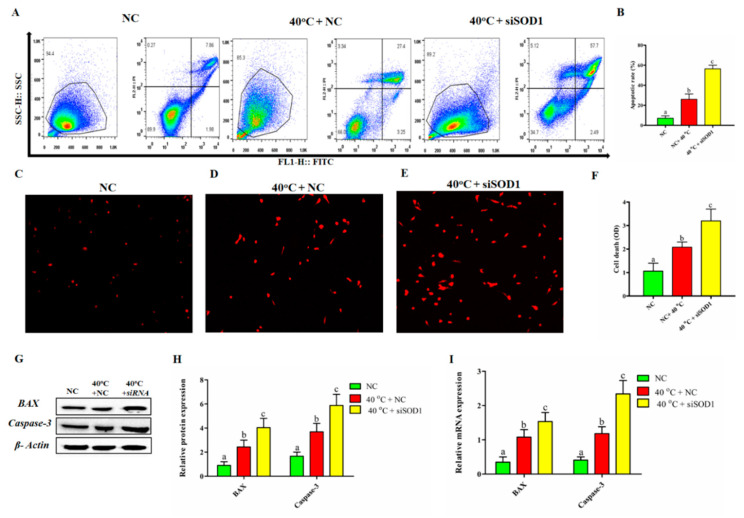
Apoptosis regulated by *SOD1* of GCs under heat stress. (**A**,**B**) Flow cytometric analysis of GCs cultured under indicated treatments (40 °C + NC, 40 °C + *siSOD1*) and corresponding NC (38 °C). (**C**–**F**) Fluorescent photomicrographs of GCs stained with Annexin V/PI FACS for indicated groups. For magnification, a 100 µm scale bar was used. Western blotting (**G**,**H**) and RT-qPCR (**I**) analysis for *BAX* and *Caspase-3* proteins expression. β-Actin and *GAPDH* was kept as housekeeping genes, respectively. Values are expressed as mean ± SEM of *n* = 3. The bars with completely distinct lettering denote a significant difference (*p* < 0.05).

The involvement of *SOD1* in the regulation of GC apoptosis under HS was explained using the Annexin VFITC kit. The flow cytometry results showed a significant increase in the apoptotic rate in the 40 °C + NC group compared with that in the NC group. Similarly, inhibiting *SOD1* in GCs at 40 °C enhanced the apoptotic rate considerably (*p* < 0.05) compared to 40 °C + NC alone (Figure 4A,B). The results of Fluorescence microscopy showed that the number of dead cells was significantly (*p* < 0.05) higher when GCs were exposed to 40 °C + NC and 40 °C +*siSOD1* compared to NC group (Figure 4C–F). In addition, *SOD1* gene regulation also altered the mRNA and protein expression of pro-apoptotic genes (*BAX* and *Caspase-3*) under HS. As shown in Figure 4G–I, compared with that in the NC group, mRNA expression and protein level of *BAX* and *Caspase-3* were significantly higher in the 40 °C + NC group. Consequently, on transcriptional and translational levels, the *si*SOD1 + 40 °C group demonstrated a substantial (*p* < 0.05) increase in *BAX* and *Caspase-3* compared to the 40 °C + NC group. Therefore, we can postulate that *SOD1* silencing promoted HS-induced cell apoptosis by activation of *BAX* and *Caspase-3*. Our findings depicted that *SOD1* has an important role in the regulation of GCs apoptosis.

### 3.6. Silencing of SOD1 Altered Cell Cycle Transition in GCs under Heat Stress

**Figure 5:**
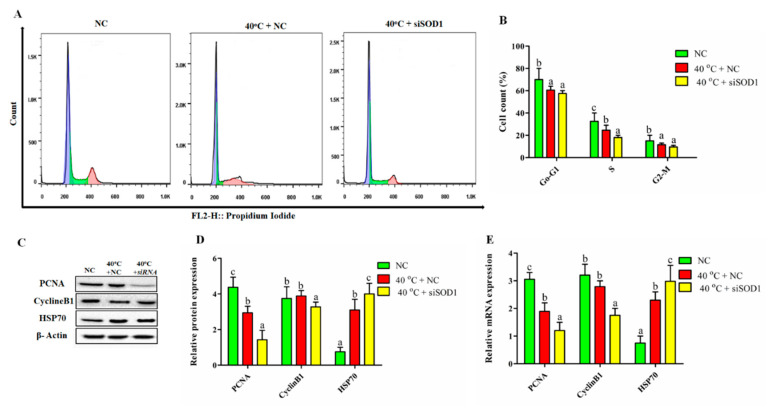
Cell cycle distribution regulated by *SOD1* in GCs under heat stress. The changes in the cell cycle in different cell treatment recorded through flow cytometric profiles groups (**A**,**B**). Western blotting was used to examine the protein expressions of *PCNA*, *CyclinB1*, and *HSP70* (**C**,**D**) and RT-qPCR (**E**). *β-Actin* and *GAPDH* were kept as housekeeping genes, respectively. Values are shown as mean ± SEM of *n* = 3. The bars with completely distinct lettering denote a significant difference (*p* < 0.05).

To check whether *SOD1* is involved in the mediation of cell proliferation under the influence of HS, flow cytometry was performed to examine the cell-cycle profile. The cell cycle distributions were estimated at the indicated groups (40 °C + NC, 40 °C + *siSOD1* and NC). Figure 5A,B demonstrated that after exposure of GCs to 40 °C and silencing of *SOD1* (*siSOD1*) significantly (*p <* 0.05) decreased the proportion of cells in the G0/G1 phase associated with subsequent decline in the S (DNA synthesis) phase and G2/M phase compared to NC group. The transcriptional and translational regulation of cell proliferation genes (*PCNA, CyclinB1, and HSP70*) were used to confirm the above findings. The mRNA and protein levels of *PCNA* and *CyclineB1* were significantly down-regulated while *HSP70* was up-regulated in 40 °C + NC and 40 °C +*siSOD1* groups compared with the NC group (Figure 5C–E). Collectively, these findings suggested that at the G0/G1 and G2/M phases the GCs were arrested for repairing DNA damage caused by *si*RNA. Thus, the cell cycle progression was regulated by *SOD1* under HS.

### 3.7. Disruption of Mitochondrial Membrane Potential of GCs under Heat Stress Caused by Silencing of SOD1

**Figure 6:**
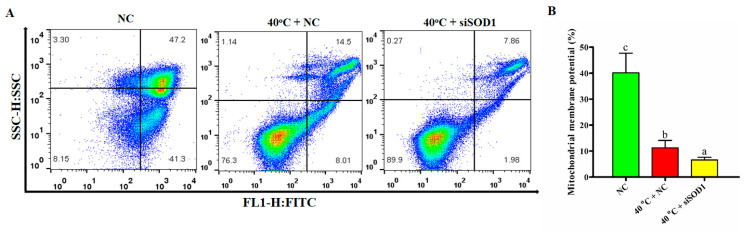
Regulation of MMP of GCs under heat stress by *SOD1*. Flow cytometric analysis of cultured GCs from NC and treated (40 °C + NC and 40 °C + *siSOD1*) groups (**A**,**B**). Values are shown as mean ± SEM of *n* = 3. The bars with completely distinct lettering denote a significant difference (*p* < 0.05).

The goal of this study was to see if *SOD1* intervention was linked to the control of the mitochondrial pathway, which is implicated in GC apoptosis under HS. Flow cytometry was used to determine the MMP of GCs. We found that The MMP was significantly (*p* < 0.05) lower in the 40 °C + NC group compared to the NC group. Likewise, the MMP was also significantly (*p* *<* 0.05) lower in 40 °C + *siSOD1* than 40 °C + NC group (Figure 6A,B). Therefore, based on these findings, we can argue that under HS, the *SOD1* has shown an essential role in the regulation of MMP in GCs.

### 3.8. Impairment of the Synthesis of P4 and E2 in GCs under Heat Stress with SOD1 Silencing

**Figure 7:**
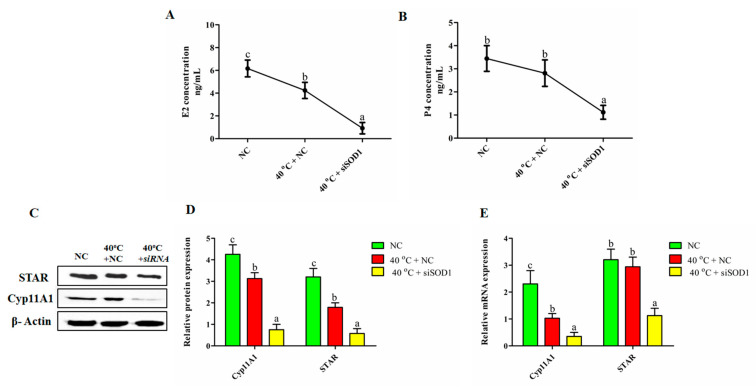
Regulation of P4 and E2 synthesis in GCs under heat stress by *SOD1.* The P4 and E2 concentrations in the culture medium were released by the GCs in different treated groups (40 °C + NC and 40 °C + *siSOD1*) and the corresponding NC group (**A**,**B**). The *STAR* and *Cyp11A1* protein expressions were analyzed by western blotting (**C**,**D**) and RT-qPCR (**E**). *β-Actin* and *GAPDH* were kept as housekeeping genes, respectively. Values are shown as mean ± SEM of *n* = 3. The bars with completely distinct lettering denote a significant difference (*p* < 0.05).

We used ELISA to estimate P4 and E2 concentrations to see how *SOD1* silencing affected hormonal shifts. Our findings established that E2 concentrations were significantly (*p* *<* 0.05) lower in 40 °C + NC group than in the NC group. Likewise, compared with 40 °C + NC group, E2 and P4 levels were significantly lower (*p* *<* 0.05) than those in 40 °C + *siSOD1* group. However, P4 did not show any significant difference with that in NC and 40 °C + NC groups (Figure 7A,B). The transcriptional and translational expression of steroidogenesis regulatory genes (*STAR* and *Cyp11A1*) further confirmed the above findings. The mRNA and protein levels of *STAR* and *Cyp11A1* were significantly down-regulated in 40 °C + NC and 40 °C + *siSOD1* groups compared to NC group (Figure 7C–E). These results confirmed that HS impaired the concentration of P4 and E2 in GCs while *SOD1* plays a key role in regulating these hormones.

## 4. Discussion

In bovine, the high environmental temperature can elevate the body temperature up to 41 °C [28]. The increase in internal body temperature related to short and long-term HS is responsible for the impaired reproductive performance of dairy cattle [31]. The deleterious effect of HS involves alterations in follicle development, impaired steroidogenesis [32], irregular follicular dynamics that affect GC function [33], disruptive oocyte maturation, fertilization, and preimplantation embryonic development [34,35]. Heat-induced is proteotoxic and results in protein denaturation that may become cytotoxic by boosting ROS production [36]. Thus, the apoptosis caused by heat stress in GCs is one of the most compromising factors affecting dairy cow fertility.

**Figure 8:**
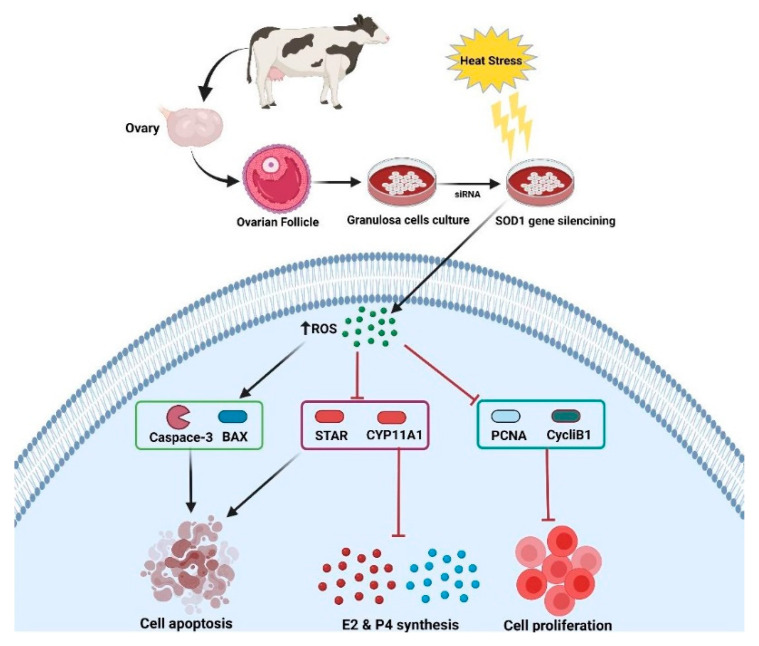
Schematic model of *SOD1* regulation of oxidative stress, apoptosis, steroidogenesis, and cell proliferation in bovine GCs exposed to HS. Reactive oxygen species (*ROS*), Bcl-2 associated x-protein (*BAX*), Proliferating cell nuclear antigen (*PCNA*), Steroidogenic acute regulatory protein (*STAR*), and Cytochrome, P450, family 11, Subfamily A, polypeptide 1 (*Cyp11A1*).

It has been well established that *SOD1* enzyme counteracts oxidative stress by converting superoxide anion radicals to O_2_ and H_2_O_2_ [25]. The present study unravels and sheds light on the noxious effects of HS on GCs viability and functions, depicting that *SOD1* plays a vital protective role against oxidative damage and apoptosis. Despite evidence for a regulation of *SOD1* under HS triggered by ROS accumulation in cells [31,37], its functional significance in heat-stressed GCs had not been investigated. In the current study, we revealed a key role of *SOD1* in regulating apoptosis and proliferation of GCs under HS (40 °C). In general, our findings verified that HS could trigger the expression of *SOD1*, while silencing or over-expression of *SOD1* significantly regulated the production of intracellular ROS, cell proliferation, apoptosis, the MMP and steroidogenesis in GCs. These findings infer that *SOD1* this gene is involved in oxidative stress in bovine GCs with a mitigation of HS-induced anomalies (Figure 8).

Our previous study showed that HS (40 °C) increases the intracellular ROS abundance, apoptosis, confirmed by the up-regulation of pro-apoptotic genes, i.e., *BAX* and *Caspase-3* [28]. Moreover, a study had documented that oxidative stress due to the increased intracellular ROS production plays a critical role in HS-induced apoptosis [38]. Consistently, the impairment of MMP function under HS has been found to be involved in mitochondrial pathway which leads to GCs apoptosis, as indicated by increases in both cleaved *Caspase-3* expression and the Bax/Bcl-2 ratio [39,40,41]. The mitochondria release Cytochrome c the cytosol when the mitochondrial function is altered. The key apoptotic program effector (*Caspase-3*) is regulated by the released cytochrome c, promoting the condensation of chromatin and fragmentation of DNA [42,43]. Interestingly, we found that the silencing of *SOD1* under HS drastically increased ROS production and apoptosis in GCs. Likewise, the altered MMP and viability in the heat-stressed *SOD1* silencing group played an important role in regulating survival and apoptosis in GCs. These findings were further manifested by the up-regulation of *BAX* and *Caspase-3* genes in the heat-stressed *SOD1* silencing group. Here, we show that silencing of *SOD1* promoted HS-mediated oxidative stress and apoptosis in GCs.

High ambient temperature is considered to have adverse effects on reproductive processes by inhibiting GCs proliferation and ovarian steroidogenesis [44,45,46,47,48]. In swine GCs, *PCNA* and *CyclinB1* are considered key proteins for cell proliferation [49,50]. *PCNA* has been considered a biological indicator for cell proliferation and is mainly regulated during cell proliferation and mainly regulated during the S phase [51]. In addition, *CyclinB1*, required for the transition of cells from G2 to M cell cycle phase, is mainly regulated during the G2/M phase of the cell cycle [52]. Biomarkers for proliferation and cell cycle such as *PCNA* and *CyclinB1* were downregulated in swine GCs under in vitro HS [47]. Our results revealed that the silencing of *SOD1* under HS to arrest in the G1 cell cycle phase. *SOD1* silencing also inhibited the proliferation of GCs, decreased the number of cells in the S phase. These results were further verified by the downregulation of *PCNA* and *CyclinB1* in the heat-stressed *SOD1* silencing group both on transcriptional and translational levels. These results suggested that *SOD1* is a critical player under HS in controlling the cellular progression in GCs.

Moreover, the regulation of genes associated with hormone synthesis (E2 and P4), such as *STAR* and *Cyp11A1* was negatively affected by HS [28]. Positive regulation of Cyp11A1 in the ovarian follicle stimulates the biosynthesis of E2 [53]. Furthermore, P4 being a steroid hormone, plays a fundamental role in bovine estrous cyclicity, and its production is associated with the positive regulation of *STAR* and *Cyp11A1* [54,55]. Some studies reported an over-secretion of ovarian hormones in porcine ovarian GCs under high temperatures [56]. In addition, it was suggested that E2 boost GCs viability by inhibiting apoptosis [21]. This is in line with the results of our previous study, which showed that HS could significantly decrease the levels of P4 and E2 in the GC culture media as well as down-regulated the expression of *STAR* and *Cyp11A1* [28]. In the current study, we found that the biosynthesis of P4 and E2 in GCs was impaired in the heat-stressed *SOD1* silencing group by down-regulating the *STAR* and *Cyp11A1* gene at transcriptional and translational levels. Thus, we can postulate that under HS, *SOD1* silencing can further aggravate apoptosis by altering the secretion of E2 and P4 in GCs. Our research can further be extended to understand the mechanism of how *SOD1* regulation can influence bovine oocyte and embryo development modulation under HS.

## 5. Conclusions

Altogether we concluded that HS induces apoptosis, alters cell proliferation, disrupts mitochondrial membrane potential, and impairs E2 and P4 synthesis in GCs by increasing intracellular ROS accumulation. Interestingly, the *SOD1* is widely expressed in bovine ovaries, and it was found to alleviate the apoptosis of GCs triggered by HS. In current study we proved that the silencing of *SOD1* under HS further deteriorated the GCs functions by promoting apoptosis and ROS generation and suppressed the proliferation of cells. This was further verified by regulating steroidogenic, associative apoptotic, and cell cycle genes (*BAX*, *Caspase-3*, *STAR*, *Cyp11A1*, *HSP70*, *PCNA*, and *CyclinB1*). These results identify a novel role of *SOD1* in the modulation of bovine ovarian GCs apoptosis, which provides a target for improving the fertility of heat-stressed dairy cows in summer.

## Figures and Tables

**Table 1:** List of gene primers used for RT-qPCR.

Gene	Accession No.	Forward 5′→3′	Reverse 5′→3′
*SOD1*	NM_201527.2	TCAATAAGGAGCAGGGACGC	AAGCCGTGTATCGTGCAGTT
*BAX*	XM_003355974.2	GGCTGGACATTGGACTTCCTTC	TGGTCACTGTCTGCCATGTGG
*Caspase-3*	XM_005671704.1	CTGGACTGTGGCATTGAGAC	GCAAAGGGACTGGAGAACC
*CyclinB1*	NM_001170768.1	AAGACGGAGCGGATCCAAAC	CCAGTGACTTCACGACCCAT
*PCNA*	NM_001291925.1	GCGTTCATAGTCGTGTTCCG	TTCAAGATGGAGCCCTGGAC
*STAR*	NM_174189.3	CCCATGGAGAGGCTTTATGA	TGATGACCGTGTCTTTTCCA
*Cyp11A1*	NM_176644.2	CTGGCATCTCCACAAAGACC	GTTCTCGATGTGGCGAAAGT
*HSP70*	NM_001014912.1	GGGGCCATGAAAACTGTTCG	TGGTGGAGATGTCTCAGGCT
*GAPDH*	NM_001034034.2	GGTGCTGAGTATGTGGTGGA	GGCATTGCTGACAATCTTGA

## Data Availability

The data presented in this study are available on request from the corresponding author.
